# Fetal size classified using gestational days rather than gestational weeks improves correlation with stillbirth risk: A statewide population study

**DOI:** 10.1371/journal.pone.0271538

**Published:** 2022-08-10

**Authors:** Natasha L. Pritchard, Stephen Tong, Susan P. Walker, Anthea C. Lindquist

**Affiliations:** 1 Department of Obstetrics and Gynaecology, University of Melbourne, Parkville, Victoria, Australia; 2 Mercy Perinatal, Mercy Hospital for Women, Heidelberg, Victoria, Australia; Hospital Sant Joan de Déu, SPAIN

## Abstract

**Objective:**

Many growth charts provide single centile cutoffs for each week of gestation, yet fetuses gain weight throughout the week. We aimed to assess whether using a single centile per week distorts the proportion of infants classified as small and their risk of stillbirth across the week.

**Design:**

Retrospective cohort study.

**Setting:**

Victoria, Australia.

**Population:**

Singleton, non-anomalous infants born from 2005–2015 (529,261).

**Methods:**

We applied growth charts to identify small-for-gestational-age (SGA) fetuses on week-based charts (single centile per gestational week) and day-based charts (centile per gestational day).

**Main outcome measures:**

Proportions <10^th^ centile by each chart, and stillbirth risk amongst SGA infants.

**Results:**

Using week-based charts, 12.1% of infants born on the first day of a gestational week were SGA, but only 7.8% on the final day; ie. an infant born at the end of the week was 44% less likely to be classed as SGA (p<0.0001). The relative risk of stillbirth amongst SGA infants born on the final day of the week compared with the first was 1.47 (95%CI 1.09–2.00, p = 0.01). Using day charts, SGA proportions were similar and stillbirth risk equal between the beginning and end of the week (9.5% vs 9.9%).

**Conclusions:**

Growth standards using a single cutoff for a gestational week overestimate the proportion of infants that are small at the beginning of the week and underestimate the proportion at the end. This distorts the risk of stillbirth amongst SGA infants based on when in the week an infant is born. Day-based charts should be used.

## Introduction

Fetal growth restriction is associated with short-term neonatal morbidity [1–6], adverse neurodevelopmental outcomes [4, 7–9], poorer adult health [10, 11], and a greater risk of stillbirth [12–15]. However, determining true growth restriction, when a fetus fails to meet its growth potential, remains clinically challenging. The most common approach to identify fetal growth restriction is to compare a fetus with its gestational aged peers, using a predetermined threshold (commonly <10^th^ centile) to identify fetuses at increased risk. These small for gestational age (<10^th^ centile) infants receive closer monitoring during pregnancy and may be subject to timed birth to reduce stillbirth risk [16–19]. Therefore, accurate classification of fetuses is important; yet substantial debate exists regarding which growth standard should be used to define these thresholds [20, 21].

Some growth standards are derived from the average birthweight (or estimated fetal weight), of all infants born (or measured) during a given gestational week [22, 23]. This means that the birthweight centile of an infant is compared with all others born within the same seven-day period. Other contemporary growth standards use statistical methods to generate smoothed centile curves, which enables the determination of birthweight centile for each individual gestational day [24–26].

Particularly in the latter part of pregnancy, a fetus can gain over 200 grams per week [22]. This means that the fetal weight on the first day of a gestational week (eg. 36 weeks +0 days) can be substantially lower than on the final day (eg. 36 weeks +6 days). If using a growth standard that reports centiles per week, rather than per day, infants may thus be assigned a lower centile if measured at the beginning of the week, and a higher centile if measured at the end of the week. The impact on perinatal outcomes of classifying fetuses as SGA by individual days or completed gestational weeks is important in determining which growth standard is optimal to stratify perinatal risk, and to assist clinicians in the interpretation of fetal size when centiles are only reported by completed weeks.

Using a large population cohort, we aimed to first quantify the impact that using a per week centile growth standard compared to a per day centile cutoff would have on the proportion of infants classified as small for gestational age. Second, we aimed to determine the relationship between stillbirth and infants classified small for gestational age using ‘week’ charts compared to ‘day’ charts.

## Methods

### Population and data collection

We conducted a retrospective cohort study using data on all infants born in Victoria, Australia, from 2005 to 2015. Data were obtained from the Consultative Council on Obstetric and Paediatric Mortality and Morbidity (CCOPMM), which is the central agency that collects and validates data on obstetric and perinatal outcomes within the state [27, 28].

Prior to data cleaning and analysis, an a priori plan was formulated to determine the inclusion and exclusion criteria, and how implausible data values would be managed. Singleton pregnancies from 24 weeks +0 days to 42 weeks +6 days’ gestation at delivery were included; pregnancies prior to 24 weeks’ were excluded due to highly variable resuscitation preferences and outcomes. Exclusion criteria included multiple pregnancy, congenital anomalies, termination of pregnancy, those with missing or implausible birthweights or missing infant sex, or where gestational age in days was not recorded.

Gestation in days was calculated using the best available measure of gestational age. This was either date of birth and estimated due date (which included ultrasound confirmation of dating if available), or date of birth and the last normal menstrual period.

Maternal height and weight data were based on that recorded at the obstetric booking visit. Parity was defined as the number of previous births (live or stillborn) over 20 weeks’ gestation. Maternal age was recorded to the nearest year at booking, and birthweight was recorded in grams. Country of birth was also self-reported. Obstetric outcome data were recorded by the attending midwife during pregnancy, delivery and after birth, using the Birthing Outcomes System. The Birthing Outcomes System is an integrated pregnancy, birth and postnatal record used by maternity hospitals throughout Victoria.

### Growth charts used

We applied Fetal Medicine Foundation fetal and neonatal (birthweight) population charts (2018) [25]. These charts were derived from both newborns, and fetuses still in utero, for a given gestational age, and so were designed to represent the entire obstetric population at any gestational age. They included all well-dated, singleton fetuses from 2011 to 2017, without congenital anomalies (95,579 infants), from two centres within the United Kingdom to derive the reference ranges [25]. Using their dataset, reference ranges were derived for both birthweight (birthweight charts) and estimated fetal weight (fetal charts). They assumed both birthweight and estimated fetal weight would have a common median and a bivariate Gaussian distribution, with the main difference being levels of spread [25]. A non-parametric quantile regression formula was used to directly estimate both birthweight and estimated fetal weight for each gestational day [29].

These charts were chosen for several reasons. First, the published charts include gestation in days, as well as gestation by completed weeks. This enabled us to compare the difference between the two, without the need for correction according to the methodology of the charts, or the population from which they were derived. Second, these charts provide both an estimated fetal weight and a birthweight centile, which enabled us to assess our findings with charts that are used in two different settings; an antenatal and a postnatal setting. Third, the study population and median birthweights have similarities to an Australian population, allowing us to approximate an appropriate 10^th^ centile, which can be a challenge when applying externally derived growth standards [22].

### Outcomes and analysis

Every newborn across all gestations was allocated a “+” number, based on the day of the gestational week that they were born; +0 being born exactly on the first day of a completed week (eg. 36+0 weeks, 252 days), through to +6 being the day before the next completed week of gestation (eg. 36+6, 258 days). This created seven groups available for analysis, with two primary groups of interest (S1 Table): i) Group +0 days contained all infants born on the first day of a completed week of gestation (24 weeks +0 days, 25+0, 26+0…. 42+0) and ii) Group +6 days contained all infants born on the final day of a completed week of gestation (24 weeks +6 days, 25+6, 26+6… 42+6).

To every infant available for analysis, we then applied the Fetal Medicine Foundation Charts in the following ways. First, we generated birthweight “week” charts; we applied the neonatal (birthweight) charts, which provide just one series of weight centiles per gestational week. This was based on the median value for that gestational week (equivalent to the exact value at +3 days for each week of the day charts). Second, we generated birthweight “day” charts: We applied the neonatal (birthweight) charts, this time using the day-by-day charts, therefore generating a specific weight centile for each completed day of gestation.

We repeated the above process using the estimated fetal weight (fetal) charts. Both fetal and birthweight charts were assessed, as fetal charts are commonly used antenatally, while birthweight charts are commonly used postnatally. This allowed us to extrapolate the results to both clinical settings.

Every infant therefore had four different birthweight centiles (birthweight week, birthweight day, estimated fetal weight week, and estimated fetal weight day centiles), and every infant was also allocated a “+” value. This allowed us to compare the impact of the four centile classifications between each of the “+” categories. In particular, we were interested in comparing the cohort of infants born at +0 days, and the cohort of infants born at +6 days, because these two cohorts were maximally affected by use of week, or day charts.

First, using week charts, we examined the proportion of infants born on each day (+0 days, +1, +2, +3, +4, +5, +6) that were classified as <10^th^ centile (small for gestational age, SGA) or <3^rd^ centile (an accepted surrogate for true fetal growth restriction). This allowed us to assess differences in classification of small infants across the week. We propose that the exact day of the gestational week that an infant was born was unlikely to have impacted the true probability of fetal growth restriction, and that differences across the week would therefore be an artefact of their classification.

We then applied day charts, and again examined the proportion of infants born on each day that were classified as <10^th^ centile or <3^rd^ centile. For each given day (+0 days to +6 days), we then assessed what proportion of infants were *reclassified* as small or large after day charts were applied, compared with week charts. This enabled us to assess the magnitude of the impact of using ‘day’ charts.

Finally, we examined stillbirth rates amongst small (<10^th^ and <3^rd^ centile) infants, focussing our comparisons on those born on +0 days and those born on +6 days. Given at +0 days an infant is at its smallest, and at +6 days its largest, these are the days on which the greatest fetal growth restriction classification error might occur. As we assumed the true probability of fetal growth restriction would occur equally on both days, we also anticipated stillbirth rates to occur equally on both days. Therefore, a different proportion of infants classified as small at +0 or +6 may impact the reported rate of stillbirth amongst small infants.

To assess this, we analysed the data in two ways. First, of those classified as <10^th^ centile by week charts, we compared the relative risk of stillbirth for infants born at +0 days with infants born at +6 days, using +0 days as the reference. We hypothesised that week charts would classify a higher proportion of infants as SGA at +0 days (when the infant is at its smallest), and the least number at +6 days (when the infant has had the full week to grow). We therefore expected the relative risk of stillbirth at +0 days to be smaller (when the SGA cohort was artificially larger and over-represented by healthy infants), and higher at +6 days (when the SGA cohort was smaller, and so represented only the smallest and highest risk infants). Second, of those classified as <10^th^ centile by day charts, we also compared the relative risk of stillbirth for infants born at +0 days to infants born at +6 days, again using +0 days as the reference. We hypothesised that day charts would classify a similar proportion of infants as SGA at both +0 days and +6 days, which would be reflected as a similar relative risk of adverse outcomes within each cohort.

We assessed these outcomes using both the birthweight and fetal standards.

### Statistical analysis

Baseline characteristics of the population were summarized by mean (standard deviation), median (25^th^– 75^th^ percentile) and number (%) according to type and distribution of the data. Relative risks were calculated and reported as point estimates with Wilson 95% confidence intervals. Significance level was two-sided, set at 0.05 and not adjusted for multiple comparisons. Statistical analysis was conducted using Stata Version 16 (StataCorp. 2019. Stata Statistical Software: Release 16.1. College Station, TX, USA).

### Ethics

Ethics approval for the project was obtained from the Mercy Health Human Research Ethics Committee (approval project number R16-10) and CCOPMM (approval number RR16-04). As this was a retrospective cohort study using de-identified data, individual patient consent was not required.

## Results

### Study population

**Fig 1:**
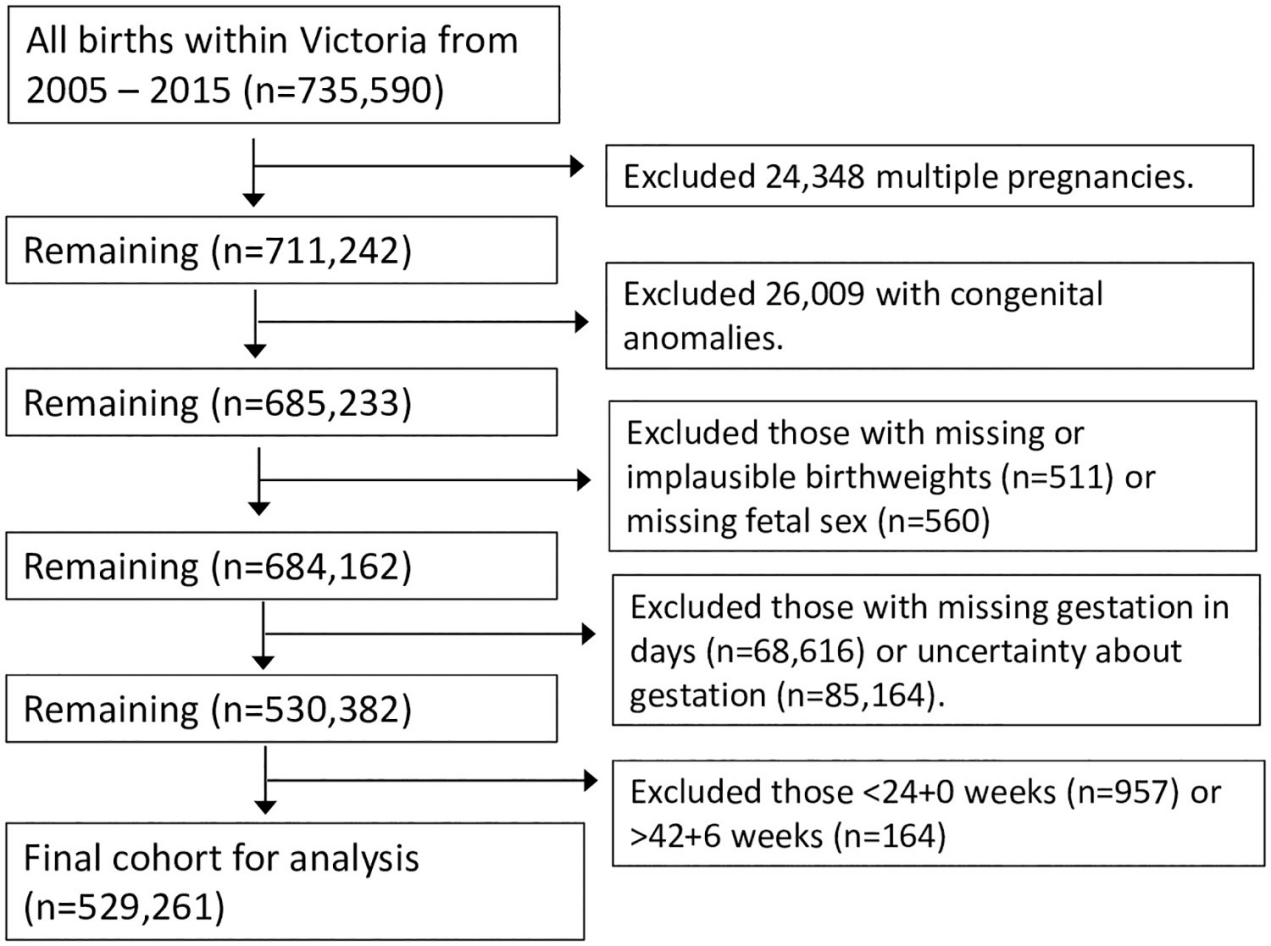
Flow diagram of exclusion criteria.

Within the study period, there were 735,590 births in Victoria. After the exclusion criteria were applied, 529,261 infants remained for final analysis (Fig 1). Baseline characteristics of those born <10^th^ centile by birthweight week and day charts, and of those born <10^th^ centile at +0 days or +6 days, are presented in Table 1. As Fetal Medicine Foundation charts are not sex-specific, a smaller proportion of male infants were considered <10^th^ centile.

**Table 1:** Baseline characteristics of those <10^th^ centile born on any day (overall), and at +0 and +6 weeks gestation, by birthweight week and day charts.

		Birthweight WEEK charts			Birthweight DAY charts		
	Total case population (n = 529,261)	*Overall cohort (n = 52*,*987)*	Infants born at +0 days (n = 9,316)	Infants born at +6 days (n = 5,275)	*Overall cohort (n = 52*,*219)*	Infants born at +0 days (n = 7,372)	Infants born at +6 days (n = 6,745)
**Birthweight**	3411 (542)	*2*,*569 (433)*	2,601 (421)	2,536 (442)	*2*,*565 (432)*	2,556 (427)	2,577 (433)
***Mean (SD)***							
**Gestational age of birth (weeks)**	39.6 (38.6–40.6)	*39*.*1 (38*.*0–40*.*1)*	39.0 (38.0–40.0)	38.9 (37.9–39.9)	*39*.*1 (38*.*0–40*.*1)*	39.0 (38.0–40.0)	38.9 (37.9–39.9)
***Median (IQR)***							
**Born <37 weeks’ gestation (%)**	28,963 (5.5)	*7*,*192 (13*.*6)*	1,067 (11.5)	831 (15.8)	*7*,*216 (13*.*8)*	826 (11.2)	1,082 (16.0)
**Male infant (%)**	269,541 (50.9)	*20*,*768 (39*.*2)*	3,698 (39.7)	2,211 (41.9)	*20*,*401 (39*.*07)*	2880 (39.1)	2704 (40.1)
**Maternal age**	30.9 (5.4)	*30*.*3 (5*.*6)*	30.4 (5.6)	30.1 (5.6)	*30*.*2 (5*.*6)*	30.4 (5.6)	30.2 (5.5)
***Mean (SD)***							
**Body Mass Index**	25.9 (5.8)	*24*.*5 (5*.*4)*	24.5 (5.3)	24.4 (5.4)	*24*.*5 (5*.*3)*	24.5 (5.3)	24.4 (5.3)
***Mean (SD)***							
**Nulliparous n (%)**	*233*,*096 (44*.*1)*	*30*,*301 (57*.*2)*	5,198 (55.8)	3,053 (57.9)	*29*,*985 (57*.*4)*	4173 (56.6)	3853 (57.1)
**Overseas born n (%)**	*166*,*089 (31*.*4)*	*21*,*415 (40*.*4)*	3,734 (40.1)	2,211 (41.9)	*21*,*168 (40*.*5)*	2961 (40.2)	2814 (41.7)

### Proportions classified as small or large by day or week birthweight charts

**Fig 2:**
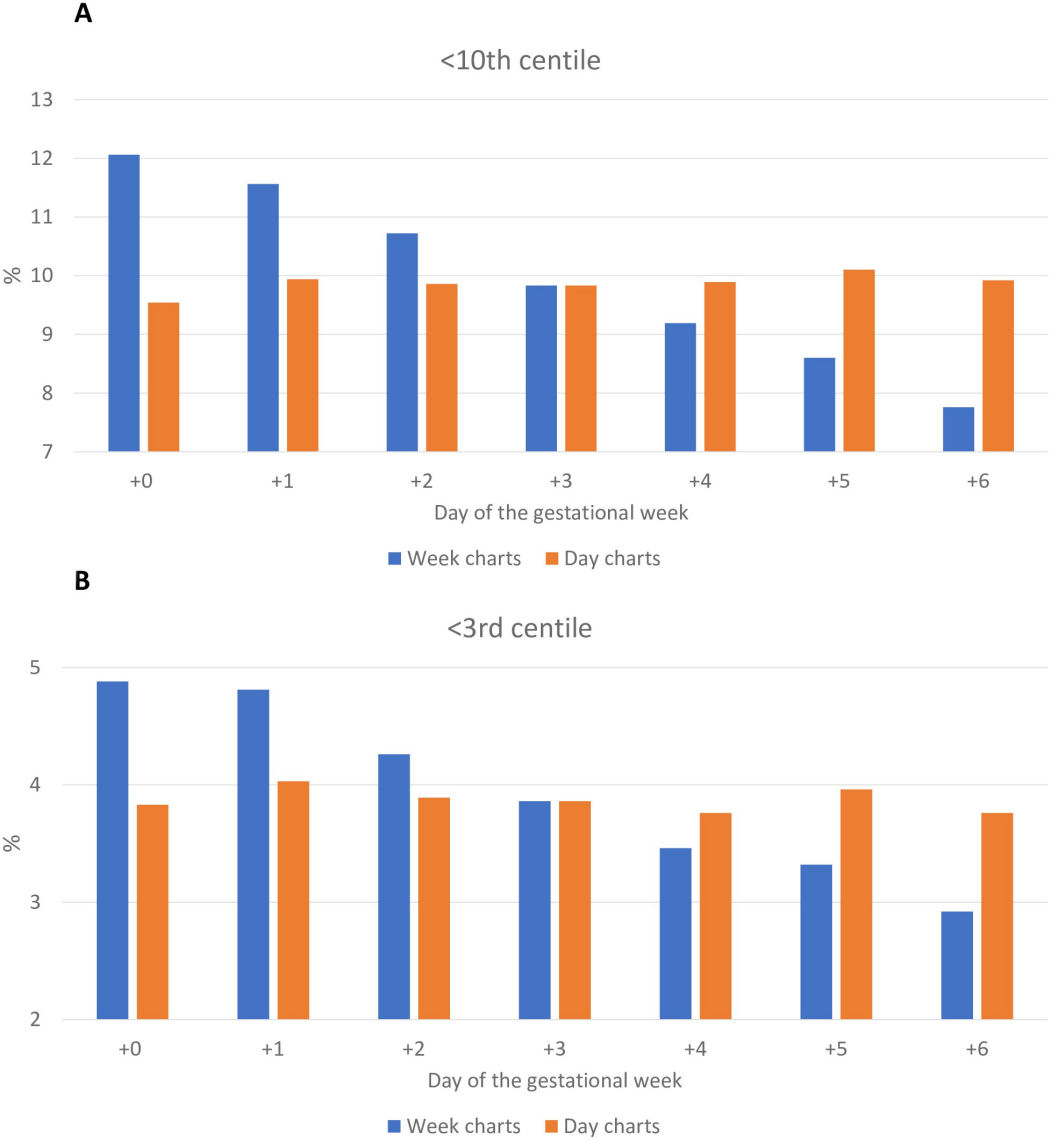
Comparison of proportions of A) <10^th^ centile and B) <3^rd^ centile based on when in the week the infant was born using birthweight week or day charts.

Of the total population, 52,987 (10.0%) newborns were classified as <10^th^ centile by birthweight week charts. Of all infants born at +0 days, week charts classified 12.1% as SGA. The proportion of infants classified as SGA dropped steadily across the gestational week cohorts to 7.8% at +6 days (Table 2, Fig 2A). The relative risk of an infant being classified as <10^th^ centile at +6 days compared with +0 days was 0.56 (95% CI 0.54–0.58, p<0.0001). This suggests that an infant is 44% less likely, by week charts, to be considered SGA if they are born at the end of the gestational week (+6 days) compared with the beginning (+0 days).

**Table 2:** Proportions of infants classified as <10^th^ centile, <3^rd^ centile and >97^th^ centile using birthweight week or day centile charts, and the relative change that application of a day centile chart causes.

	+0 days	+1 days	+2 days	+3 days	+4 days	+5 days	+6 days	Overall
	n = 77,247	n = 78,033	n = 77,936	n = 78,032	n = 77,125	n = 72,872	n = 68,016	n = 529,261
**<10**^**th**^ **CENTILE**								
**WEEKS**	9,316 (12.1)	9,017 (11.6)	8,353 (10.7)	7,674 (9.8)	7,086 (9.2)	6,266 (8.6)	5,275 (7.8)	52,987 (10.0)
**DAYS**	7,372 (9.5)	7,758 (9.9)	7,683 (9.9)	7,674 (9.8)	7,628 (9.9)	7,359 (10.1)	6,745 (9.9)	52,219 (9.9)
***Change from week to day charts (%)***	*-20*.*9*	*-14*.*0*	*-8*.*0*	*0*	*+7*.*64*	*+17*.*4*	*+27*.*9*	*-0*.*1*
**<3**^**rd**^ **CENTILE**								
**WEEKS**	3,773 (4.9)	3,753 (4.8)	3,323 (4.3)	3,011 (3.9)	2,667 (3.5)	2,416 (3.3)	1,986 (2.9)	20,929 (4.0)
**DAYS**	2,961 (3.8)	3,141 (4.0)	3,030 (3.9)	3,011 (3.9)	2,902 (3.8)	2,887 (4.0)	2,556 (3.8)	20,488 (3.9)
***Change from week to day charts (%)***	*-21*.*5*	*-16*.*3*	*-8*.*8*	*0*	*+8*.*8*	*+19*.*5*	*+28*.*8*	*-0*.*1*

Overall, 52,219 (9.9%) newborns were classified as <10^th^ centile by birthweight day charts. Using day charts, the proportion classified as <10^th^ centile was similar across the gestational week; with 9.5% <10^th^ centile at the beginning (+0 days), and 9.9% at the end (+6 days) (Table 2, Fig 2A).

We then assessed the change in classification if day charts were used compared with week charts. When day charts were applied instead of week charts, 20.9% fewer infants born at +0 days were considered <10^th^ centile (Table 2). When day charts were applied instead of week charts, 27.9% more infants born at +6 days were considered <10^th^ centile.

The same findings were seen in those infants born <3^rd^ centile (Table 2, Fig 2B). Using week charts, 4.9% born at +0 days were considered <3^rd^ centile, dropping to 2.9% at +6 days. The relative risk of being considered <3^rd^ centile at +6 days compared with +0 days was 0.60 (95% CI 0.57–0.63), suggesting that an infant is 40% less likely to be considered <3^rd^ centile on week charts, if born at the end of the gestational week compared with the beginning. When day charts were used, the proportions classified <3^rd^ centile were equivalent at +0 days and +6 days (3.8% vs 3.8%).

### Stillbirth risk

Overall, there was no difference in the rate of stillbirth between all infants born at +0 days (197 stillbirths; 0.26% of total births) and those born at +6 days (196 stillbirths; 0.29% of total births) (p = 0.23). We then examined stillbirth rates amongst those classified as <10^th^ centile by week charts (Table 3). The risk of stillbirth in the SGA cohort (<10^th^ centile) born at the beginning of the week (+0) was used as our reference, and we compared it with the relative risk of stillbirth in the SGA cohort born at the end of the week (+6). Using week charts, there was a higher relative risk of stillbirth amongst SGA infants at +6 compared with +0 (RR 1.47, 95% CI 1.09–2.00). When day charts were used, there was no significant difference in the stillbirth risk between the +0 days and +6 days cohorts (Table 3).

**Table 3:** Stillbirth rates, and relative risk of stillbirth at +6 days compared to +0 days by day and week charts, using birthweight standards.

	Week charts		Day charts	
	+0 days	+6 days	+0 days	+6 days
**<10**^**TH**^ **CENTILE**				
**Total <10**^**th**^ **centile n (%)**	9,316 (12.1)	5,275 (7.8)	7,372 (9.5)	6,745 (9.9)
**Stillborn n =**	95	79	83	94
**Stillbirth rate/ 1000**	10.2	15.0	11.3	13.9
**Relative risk (95% CI)**	*Ref*	*1*.*47 (1*.*09–2*.*00)*, *p = 0*.*01*	*Ref*	*1*.*24 (0*.*92–1*.*66)*, *p = 0*.*15*
**<3**^**RD**^ **CENTILE**				
**Total <3**^**rd**^ **centile n (%)**	3,773 (4.9)	1,986 (2.9)	2,961 (3.8)	2,556 (3.8)
**Stillborn n =**	69	63	62	68
**Stillbirth rate/ 1000**	18.3	31.7	20.9	26.6
**Relative risk (95% CI)**	*Ref*	*1*.*73 (1*.*24–2*.*43)*, *p = 0*.*0012*	*Ref*	*1*.*27 (0*.*90–1*.*79)*, *p = 0*.*17*

When a cutoff of <3^rd^ centile was applied, an even stronger trend was observed (Table 3). Using week charts, the relative risk of stillbirth amongst those born at +6 days was higher than at +0 days (RR 1.73, 95% CI 1.24–2.43). If day charts were used, no significant difference in the risk of stillbirth was seen between the two cohorts.

We replicated our analysis, using the estimated fetal weight standard instead of the birthweight standard, to ensure that the same effect size was seen using a fetal growth standard (which has reduced variation around the mean). The same findings were seen, with significantly increased stillbirth risk in small infants born at the end of the week (Table 4).

**Table 4:** Stillbirth rates, and relative risk of stillbirth at +6 compared to +0 by day and week charts, using estimated fetal weight standards.

	Week charts		Day charts	
	+0 days	+6 days	+0 days	+6 days
**<10**^**TH**^ **CENTILE**				
**Total <10**^**th**^ **centile**	13,906	8,400	11,368	10410
**Stillborn**	106	93	93	107
**Stillbirth rate/ 1000**	7.6	11.1	8.2	10.3
**RR (95% CI)**	*Ref*	*1*.*45 (1*.*10–1*.*92)*, *p = 0*.*008*	*Ref*	*1*.*26 (0*.*95–1*.*66)*, *p = 0*.*11*
**<3**^**RD**^ **CENTILE**				
**Total <3**^**rd**^ **centile**	7305	4043	5843	5289
**Stillborn**	88	74	82	78
**Stillbirth rate/ 1000**	12.0	18.3	14.0	14.7
**RR (95% CI)**	*Ref*	*1*.*52 (1*.*12–2*.*06)*, *p = 0*.*007*	*Ref*	*1*.*15 (0*.*84–1*.*56) p = 0*.*38*

## Discussion

### Main findings

In a large, population-based cohort study, we compared the impact of using growth standards with a common centile cutoff across the gestational week (‘week’ charts) vs growth standards applying a unique centile cutoff for each gestational day (‘day’ charts). Using week charts, we found that an infant was almost half as likely to be considered <10^th^ centile if born at the beginning of the week (+0 days) compared with the end (+6 days). In relative terms, this meant 21% fewer infants were <10^th^ centile at +0 days, and 28% more at +6 days, highlighting a substantial reclassification These same findings were demonstrated for those classified <3^rd^ centile—the cohort most likely to be subject to obstetric interventions based purely on size [30]. Importantly, whether day or week charts were used then impacted the relative risk of stillbirth amongst small for gestational age infants born at +0 days and +6 days.

When week charts were used, the <10^th^ centile proportion was highest at +0 days, but the relative risk of stillbirth lowest. This suggests that the SGA cohort at the beginning of the week has been diluted by small, healthy infants, that have not yet had the benefit of the seven-day period in which to grow. When day charts were used, and equivalent proportions were considered SGA, no significant differences in stillbirth risk were seen between +0 days and +6 days. This suggests that a measurable misclassification is occurring when week charts are used, which is corrected by using day charts.

The magnitude of these classification differences in SGA has important clinical implications for those infants born, or measured, at the extremes of a gestational week. If an infant is identified as <10^th^, and particularly <3^rd^ centile, it is treated as increasing the pre-test probability of that infant having true fetal growth restriction [31]. Fetal weight centile therefore forms an important part of all antenatal surveillance regimes, including decisions regarding time and mode of birth [32, 33]. Thus, it is important that the classification of smallness is applied consistently across the gestational week and correlates with perinatal risk. If an infant is born at 37 weeks +6 days, the likelihood of that infant being considered <10^th^ centile should not be half that of if it was born a single day later, at 38 weeks +0 days, with the risk of stillbirth- if small- altered by almost 50%.

### Strengths and limitations

The large size of our statewide cohort has allowed us to robustly assess the impact of using day specific growth standards on the detection of FGR and its most important obstetric outcome, stillbirth. As the first study to directly quantify the impact on stillbirth of using day charts over week charts, our findings make a useful contribution to the field by proposing a simple and effective way to improve classification of fetuses at risk. Our study is limited by its retrospective design, and by the need to exclude cases that did not have gestation in days available. However, there is no reason to suspect this missing data would have been unequal between groups, and it is therefore unlikely to have impacted the results. Another limitation is that we were only able to apply growth standards to a population of infants already born. This means that we are only able to hypothesise about the potential benefit of adjusting growth standards to day-specific cutoffs on antenatal management decisions, informed by ultrasound estimated fetal weights.

### Interpretation

There is considerable debate about which growth standard should be used to define size in obstetric practice [20]. Many aspects of growth standards are highly controversial such as customisation on maternal characteristics [34, 35], with unresolved debate regarding which physiological characteristics have likely pathological influences on fetal growth. Here, we have instead focussed on providing evidence of benefit for one specific aspect of growth standards- the use of ‘day’, rather than ‘week’ -charts, which appear to more accurately classify fetal risk in both the clinical and research settings.

Although many contemporary growth standards do provide the option for an individual centile for each gestational day [34, 36–38], others rely only on a single set of centiles for each completed week. Several international charts in use [23, 39], and the standards currently used in Australia for postnatal birthweight classification [22, 40], provide only a centile cutoff for each week. This means many clinicians still receive ultrasound or birthweight reports derived from a single weekly centile cutoff. If a week-based growth chart is used, it would still be necessary to apply the correct gestational weekly centile cutoff. However, our findings provide evidence that the exact day within a gestational week that an infant is measured should be taken into consideration when interpreting the centile thresholds.

Moreover, for many years, there has been increasing concern about the high rates of obstetric intervention and the potential for iatrogenic harm [41–43]. Here, we provide a simple method by which SGA classification can be improved to correlate better with perinatal outcomes without inflating the proportion of pregnancies considered at risk.

### Conclusions

Using growth standards that rely on a common centile cutoff for every day of a given gestational week, an infant is significantly more likely to be considered small if born earlier in the gestational week, and significantly less likely if born at the end of the week. This leads to an artefactual distortion of the SGA cohorts, and unequal distribution of relative risk of stillbirth by days of the week. This anomaly can be overcome by the use of charts that provide a centile cutoff for each day of the week, producing uniform SGA classification across the week, and correcting the distorted risk of stillbirth. We therefore recommend use of growth standards that give an individual centile cutoff for each day of the week. If this is not practical, we urge clinicians to be cognisant of the timing of assessment within the week when making management decisions.

## Supporting information

S1 Table Classification of infants into “+” categories—All infants born on any day of a gestational week were grouped together for analysis. (DOCX)
