# Supplementary material for: Fetal size classified using gestational days rather than gestational weeks improves correlation with stillbirth risk: A statewide population study
Source: PLoS One. 2022 Aug 10;17(8):e0271538. doi: 10.1371/journal.pone.0271538 (PMC9365147; doi:10.1371/journal.pone.0271538)
Supplement: S1 Table — (DOCX) [file pone.0271538.s001.docx]

**S1 Table: Classification of infants into “+” categories – all infants born on any day of a gestational week were grouped together for analysis**

| **+0 days** | **+1 days** | **+2 days** | **+3 days** | **+4 days** | **+5 days** | **+6 days** |
| --- | --- | --- | --- | --- | --- | --- |
| 24+0 | 24+1 | 24+2 | 24+3 | 24+4 | 24+5 | 24+6 |
| 25+0 | 25+1 | 25+2 | 25+3 | 25+4 | 25+5 | 25+6 |
| 26+0 | 26+1 | 26+2 | 26+3 | 26+4 | 26+5 | 26+6 |
| 27+0 | 27+1 | 27+2 | 27+3 | 27+4 | 27+5 | 27+6 |
| 28+0 | 28+1 | 28+2 | 28+3 | 28+4 | 28+5 | 28+6 |
| 29+0 | 29+1 | 29+2 | 29+3 | 29+4 | 29+5 | 29+6 |
| 30+0 | 30+1 | 30+2 | 30+3 | 30+4 | 30+5 | 30+6 |
| 31+0 | 31+1 | 31+2 | 31+3 | 31+4 | 31+5 | 31+6 |
| 32+0 | 32+1 | 32+2 | 32+3 | 32+4 | 32+5 | 32+6 |
| 33+0 | 33+1 | 33+2 | 33+3 | 33+4 | 33+5 | 33+6 |
| 34+0 | 34+1 | 34+2 | 34+3 | 34+4 | 34+5 | 34+6 |
| 35+0 | 35+1 | 35+2 | 35+3 | 35+4 | 35+5 | 35+6 |
| 36+0 | 35+1 | 35+2 | 35+3 | 35+4 | 35+5 | 35+6 |
| 37+0 | 37+1 | 37+2 | 37+3 | 37+4 | 37+5 | 37+6 |
| 38+0 | 38+1 | 38+2 | 38+3 | 38+4 | 38+5 | 38+6 |
| 39+0 | 39+1 | 39+2 | 39+3 | 39+4 | 39+5 | 39+6 |
| 40+0 | 40+1 | 40+2 | 40+3 | 40+4 | 40+5 | 40+6 |
| 41+0 | 41+1 | 41+2 | 41+3 | 41+4 | 41+5 | 41+6 |
| 42+0 | 42+1 | 42+2 | 42+3 | 42+4 | 42+5 | 42+6 |
